# Clinical characteristics, radiological features, and disease severity of bronchiectasis according to the spirometric pattern

**DOI:** 10.1038/s41598-022-17085-3

**Published:** 2022-08-01

**Authors:** Sun-Hyung Kim, Bumhee Yang, Jin Young Yoo, Jun Yeun Cho, Hyeran Kang, Yoon Mi Shin, Eung-Gook Kim, Ki Man Lee, Kang Hyeon Choe

**Affiliations:** 1 Division of Pulmonary and Critical Care Medicine, Department of Internal Medicine, Chungbuk National University Hospital, Chungbuk National University College of Medicine, Cheongju, Korea; 2 Department of Radiology, Chungbuk National University Hospital, Chungbuk National University College of Medicine, Cheongju, Korea; 3 Department of Biochemistry, Chungbuk National University College of Medicine, Cheongju, Korea

**Keywords:** Cystic fibrosis, Risk factors

## Abstract

Bronchiectasis show various ventilatory disorders in pulmonary function. The characteristics and severity of patients with bronchiectasis according to these pulmonary dysfunctions are still very limited. This study aimed to evaluate the clinical, radiologic feature and the disease severity of patients with bronchiectasis according to spirometric patterns. We retrospectively evaluated 506 patients with bronchiectasis who underwent pulmonary lung function test (PFT) at a referral hospital between 2014 to 2021. The results showed that cylindrical type was the most common (70.8%) type of bronchiectasis on chest Computed tomography (CT), and 70% of patients had bilateral lung involvement. On the other hand, obstructive ventilatory disorder was the most common (51.6%), followed by normal ventilation (30%) and restrictive ventilatory disorder (18.4%). The modified Medical Research Council (mMRC) was highest in patients with obstructive ventilatory disorders, Modified Reiff score [median (interquartile range)] [6 (3–10), P < 0.001], FACED (FEV_1_, Age, Chronic colonization, Extension, and Dyspnea) score [3 (1–4), P < 0.001], and Bronchiectasis Severity (BSI) score [8 (5–11), P < 0.001] showed significantly highest values of obstructive ventilatory disorder rather than restrictive ventilatory disorder and normal ventilation. More than half of patients with bronchiectasis had obstructive ventilatory disorder. Bronchiectasis with obstructive ventilatory disorders has more dyspnea symptom, more disease severity and more radiologic severity. There was no significant association between spirometric pattern and radiologic type, but the more severe the radiologic severity, the more severe the lung function impairment.

## Introduction

Non-cystic fibrosis bronchiectasis (hereafter referred to as bronchiectasis) is a chronic respiratory disease characterized by permanent dilatation of the bronchi and chronic respiratory symptoms, such as cough, expectoration of sputum, and dyspnea. The inherent dysfunction in mucociliary clearance leads to persistent bacterial infection, chronic inflammation of the bronchial tree, and progressive tissue destruction. Thus, persistent airway destruction leads to decreased lung function and respiratory failure^1,2^.

Previous studies have reported that the morphological changes in bronchiectasis are associated with pulmonary dysfunction^3–5^. Research suggests that the development of bronchiectasis is promoted by the early involvement of the lymphoid follicles in the small airways, which gradually leads to the obstruction of the more distal airways^6^. Accordingly, it has been known that bronchiectasis is accompanied by airflow limitation^7^. As a result, forced expiratory volume in 1 s (FEV1) was used to evaluate pulmonary dysfunction in patients with bronchiectasis, as a same line, FEV_1_ was included in Bronchiectasis Severity Index (BSI) score and FACED (FEV_1_, Age, Chronic colonization, Extension, and Dyspnea) score to evaluate the severity and prognosis of patient with bronchiectasis^8,9^. Moreover, recent studies have reported that patients with bronchiectasis exhibit various ventilatory disorders on pulmonary function tests, such as obstructive, restrictive, and mixed ventilatory disorder^4,10–12^. Although various ventilatory disorders of bronchiectasis have been reported as described above, studies on the characteristics and severity of patients with bronchiectasis according to these pulmonary dysfunctions are still very limited.

Hence, we investigated the clinical and radiological features of patients with bronchiectasis according to the spirometric pattern. Moreover, we further evaluated the disease severity in patients with bronchiectasis according to spirometric patterns.

## Results

### Baseline characteristics

The baseline characteristics of 506 patients with bronchiectasis are presented in Table 1. The median age was 66 years interquartile range (IQR 53–79 years), and 254 (50.2%) patients were male. The median body mass index (BMI) was 23.0 (IQR 19.3–26.7). Current and ex-smokers accounted for 34.8% of the study population, and 23.7% of patients had a history of tuberculosis. The distribution of comorbidities in patients with bronchiectasis was as follows: chronic obstructive pulmonary disease (COPD) (51.6%), followed by cardiovascular disease (37.7%), asthma (16.2%), and diabetes mellitus (12.6%). Cylindrical bronchiectasis was the most common (70.8%) morphology on chest CT, and 70% of patients exhibited bilateral lung involvement. The pulmonary function parameters were as follows: forced vital capacity (FVC) % predicted, 84% (IQR 70–95); FEV_1_/FVC, 73% (IQR 56–90); and FEV1% predicted, 69% (IQR 55–78). Microorganisms were identified in 32.4% of patients: *Pseudomonas aeruginosa* (18.0%) was the most frequently identified species, followed by *Klebsiella pneumoniae* (8.5%), and *Staphylococcus aureus* (4.9%). NTM were identified in 10.1% of patients, of which *Mycobacterium avium* complex (MAC) was the most common type (8.3%).

**Table 1:** Baseline characteristics of patients with bronchiectasis.

Variables	Total
	N = 506
Age, years	66 (53–79)
Sex, male	254 (50.2)
BMI (kg/m^2^)	23.0 (19.3–26.7)
**Smoking history**	
Current or ex-smoker	176 (34.8)
Previous history of TB	120 (23.7)
Previous history of pertussis	15 (3.0)
**Comorbidities**	
COPD	261 (51.6)
Asthma	82 (16.2)
Cardiovascular disease	191 (37.7)
Diabetes mellitus	64 (12.6)
Chronic liver disease	16 (3.2)
Chronic kidney disease	15 (3.0)
Chronic cavitary pulmonary aspergillosis	10 (2.0)
**Bronchiectasis type**	
Cylindrical	358 (70.8)
Varicose	12 (2.4)
Cystic	315 (62.3)
**Lung involvement**	
One lung	152 (30.0)
Both lungs	354 (70.0)
**Spirometry patterns**	
FVC, L	2.5 (2.0–3.3)
FVC, % predicted	84 (70–95)
FEV_1_, L	1.7 (1.2–2.2)
FEV_1_, % predicted	73 (56–90)
FEV_1_/FVC, %	69 (55–78)
**Microbiology**	164 (32.4)
*Pseudomonas aeruginosa*	91 (18.0)
*Haemophilus influenzae*	12 (2.4)
*Staphylococcus aureus*	25 (4.9)
*Klebsiella pneumoniae*	43 (8.5)
*Streptococcus pneumoniae*	12 (2.4)
Others^a^	52 (10.3)
**Non-tuberculous mycobacteria**	50 (10.1)
MAC	42 (8.3)
MABC	8 (1.6)

Data are presented as the median (interquartile range) or numbers (%).

*BMI* body mass index, *TB* tuberculosis, *COPD* chronic obstructive pulmonary disease, *FVC* forced vital capacity, *FEV*_*1*_ forced expiratory volume in 1 s, *MAC* *Mycobacterium avium-intracellulare* complex, *MABC* *M. abscessus* complex.

^a^Others had multidrug-resistant bacteria identified in hospital-acquired pneumonia (e.g., *Acinetobacter baumannii, Stenotrophomonas maltophilia*).

### Clinical characteristics according to the spirometric pattern

As shown in Table 2, 152 patients (30.0%) with bronchiectasis had normal ventilation, 261 patients (51.6%) had obstructive ventilatory disorder, and 93 patients (18.4%) had restrictive ventilatory disorder. The median ages of patients with normal ventilation, obstructive ventilatory disorder, and restrictive ventilatory disorder were 66 (IQR 58–72), 68 (IQR 62–75), and 64 (IQR 56–71) years, respectively (*P* < 0.001). The proportion of males was highest in patients with obstructive ventilatory disorder, followed by those with normal ventilation and those with restrictive ventilatory disorder (62.1%, 41.4%, and 31.2%, respectively, *P* < 0.001). The proportion of current- or ex-smokers was higher among patients with obstructive ventilatory disorders compared to that in patients with normal ventilation or restrictive ventilatory disorders (43.7%, 28.3%, and 20.4%, respectively, *P* < 0.001). The prevalence of COPD (100%), asthma (21.8%), cardiovascular disease (43.7%), and diabetes mellitus (16.9%) was the highest in patients with obstructive ventilatory disorder from amongst the entire study population. Most of the microorganisms were cultured in the obstructive ventilatory disorder, followed by restrictive ventilatory disorder and normal ventilation. (41.4%, 32.3% and 15.8%, respectively, *P* < 0.001). The frequency of culturing for *P. aeruginosa* (23.8%, 17.2% and 8.6%, respectively, *P* < 0.001), and others (10.3%, 6.5% and 1.3%, respectively, *P* < 0.001) was significantly higher in the obstructive ventilatory disorder and restrictive ventilatory disorder than that in the normal ventilation. The prevalence of NTM (20.4%) was the highest in patients with restrictive ventilatory disorder from amongs the entire study population.

**Table 2:** Baseline characteristics of patients with bronchiectasis according to spirometric pattern.

Variables	Normal	Obstructive	Restrictive	P-value
	N = 152	N = 261	N = 93	
Age, years	66 (58–72)	68 (62–75)^a,c^	64 (56–71)^c^	< 0.001
Sex, male	63 (41.4)	162 (62.1)^a,c^	29 (31.2)^c^	< 0.001
BMI (kg/m^2^)	23.4 (21.1–25.5)	22.9 (20.4–25.2)	22.5 (19.9–25.6)	0.275
**Smoking history**				
Current or ex-smoker	43 (28.3)	114 (43.7)^a,c^	19 (20.4^)c^	< 0.001
Previous history of TB	29 (19.1)	68 (26.1)	23 (24.7)	0.266
Previous history of pertussis	4 (2.6)	5 (1.9)	6 (6.5)	0.083
**Comorbidities**				
COPD	0	261 (100)^a,c^	0^c^	< 0.001
Asthma	20 (13.2)	57 (21.8)^a,c^	5 (5.4)^c^	0.001
Cardiovascular disease	46 (30.3)	114 (43.7)	31 (33.3)	0.016
Diabetes mellitus	13 (8.6)	44 (16.9)^a,c^	7 (7.5)^c^	0.013
Chronic liver disease	4 (2.6)	8 (3.1)	4 (4.3)	0.763
Chronic kidney disease	1 (0.7)	10 (3.8)	4 (4.3)	0.131
Chronic cavitary pulmonary aspergillosis	0	7 (2.7)	3 (3.2)	0.053
**Spirometry**				
FVC, L	2.9 (2.4–3.5)	2.5 (1.9–3.3)^a,c^	2.0 (1.6–2.5)^b,c^	< 0.001
FVC, % predicted	94 (87–102)	82 (68–94)^a,c^	68 (59–74)^b,c^	< 0.001
FEV_1_, L	2.2 (1.9–2.7)	1.3 (1.0–1.8)^a,c^	1.6 (1.2–2.1)^b,^^c^	< 0.001
FEV_1_, % predicted	98 (88–108)	61 (45–74)^a,c^	69 (61–79)^b,c^	< 0.001
FEV_1_/FVC, %	78 (74–81)	56 (48–64)^a,c^	79 (74–86)^c^	< 0.001
**Microbiology**	24 (15.8)	108 (41.4)^a^	30 (32.3)^b^	< 0.001
*Pseudomonas aeruginosa*	13 (8.6)	62 (23.8)^a^	16 (17.2)^b^	0.001
*Haemophilus influenzae*	1 (0.7)	9 (3.4)	2 (2.2)	0.196
*Staphylococcus aureus*	3 (2.0)	18 (6.9)^a^	4 (4.3)	0.080
*Klebsiella pneumoniae*	9 (5.9)	26 (10.0)	8 (8.6)	0.364
*Streptococcus pneumoniae*	1 (0.7)	9 (3.4)	2(2.2)	0.196
Others^a^	2 (1.3)	27 (10.3)^a^	6 (6.5)^b^	0.002
**Non-tuberculous mycobacteria**	15 (9.9)	17 (6.5)^c^	19 (20.4)^b,c^	0.001
MAC	13/15 (86.7)	15/17 (88.2)	14/19 (73.7)	0.454
MABC	2/15 (13.3)	2/17 (11.8)	4/19 (21.1)	0.167

Data are presented as the median (interquartile range) or numbers (%).

One patient had co-infection with MAC and *M. abscessus.*

*BMI* body mass index, *COPD* chronic obstructive pulmonary disease, *TB* tuberculosis, *FVC* forced vital capacity, *FEV*_*1*_ forced expiratory volume in 1 s, *NTM* nontuberculous mycobacteria, *MAC* *Mycobacterium avium-intracellulare* complex, *MABC* *M. abscessus* complex.

^a^Others had multidrug-resistant bacteria identified in hospital-acquired pneumonia (e.g., *Acinetobacter baumannii, Stenotrophomonas maltophilia*).

Due to Bonferroni correction with three comparisons, the P-value of 0.05 corresponds to 0.17 (0.05/3).

^a^There was a significant difference between normal lung function and the obstructive pattern.

^b^There was a significant difference between normal lung function and the restrictive pattern.

^c^There was a significant difference between the obstructive and restrictive patterns.

### Respiratory symptoms, radiological features, and disease severity scales in patients with bronchiectasis according to the spirometric pattern

As shown in Table 3, the modified Medical Research Council (mMRC) was the highest in patients with obstructive ventilatory disorder, followed by those with restrictive ventilatory disorder and normal ventilation [median (IQR)] [1 (1–2), 1 (0–2), and 0 (0–1), respectively; *P* < 0.001]. The modified Reiff score [6 (3–10), *P* < 0.001)]; FACED score [3 (1–4), *P* < 0.001], and BSI score [8 (5–11), *P* < 0.001] were significantly higher in the obstructive ventilatory disorder group than those in the restrictive ventilatory disorder and normal ventilation groups. There was no significant correlation between the lung function pattern and bronchiectasis type. The proportion of bilateral lung involvement was the highest in patients with obstructive ventilatory disorder, followed by those with restrictive ventilatory disorder and normal ventilation (79.7%, 72.0%, and 52.0%, respectively, *P* < 0.001). The disease severity indices, i.e., the BSI and FACED scores, were the highest in patients with obstructive ventilatory disorder, followed by those with restrictive ventilatory disorder and normal ventilation [8 (5–11), 6 (4–9) and 4 (2–7), respectively, *P* < 0.001 for the BSI score; 3 (1–4), 1 (1–3) and 1 (0–2), respectively, *P* < 0.001 for the FACED score].

**Table 3:** Respiratory symptoms, radiological features and disease severity scales of patients with bronchiectasis according to spirometric patterns.

Variables	Total N = 506	Normal N = 152	Obstructive N = 261	Restrictive N = 93	P-value
mMRC	0 (1–2)	0 (0–1)	1 (1–2)^a,c^	1 (0–2)^b,c^	< 0.001
Hemoptysis	150 (29.6)	45 (29.6)	79 (30.3)	26 (28.0)	0.916
BAE	36/150 (24.0)	8/45 (17.8)	20/79 (25.3)	8/26 (30.8)	0.431
Modified Reiff score	5 (3–8)	3 (2–6)	6 (3–10)^a^	5 (3–8)^b^	< 0.001
**Bronchiectasis type**					
Cylindrical	358 (70.8)	106 (69.7)	185 (70.9)	67 (72.0)	0.926
Varicose	12 (2.4)	3 (2.0)	7 (2.7)	2 (2.2)	0.890
Cystic	315 (62.3)	87 (57.2)	167 (64.0)	61 (65.6)	0.301
**Lung involvement**					
One lung	152 (30.0)	73 (48.0)	53 (20.3)^a^	26 (28.0) ^b^	< 0.001
Both lungs	354 (70.0)	79 (52.0)	208 (79.7)^a^	67 (72.0)^b^	< 0.001
FACED score	2 (1–3)	1 (0–2)	3 (1–4)^a,c^	1 (1–3)^b,c^	< 0.001
**FACED score risk class**					< 0.001
Mild (0–2)	303 (59.9)	122 (80.3)	114 (43.7)^a,c^	67 (72.0)^b,c^	
Moderate (3–4)	158 (31.2)	30 (197)	106 (40.6)^a,c^	22 (23.7)^b,c^	
Severe (5–7)	45 (8.9)	0	41 (15.7)^a,c^	4 (4.3)^b,c^	
BSI score	6 (4–10)	4 (2–7)	8 (5–11)^a,c^	6 (4–9)^b,c^	< 0.001
**BSI score risk class**					< 0.001
Mild (0–4)	179 (35.4)	80 (52.6)	63 (24.1)^a,c^	36 (38.7)^b,c^	
Moderate (5–8)	211 (41.7)	62 (40.8)	109 (41.8)^a,c^	40 (43.0)^b,c^	
Severe (9+)	116 (22.9)	10 (6.6)	89 (34.1)^a,c^	17 (18.3)^b,c^	
Follow-up duration, months	28 (6–55)	17 (2–42)	36 (12–69)^a,c^	17 (4–43) ^c^	< 0.001

Data are presented as the median (interquartile range) or numbers (%).

Due to Bonferroni correction with three comparisons, the P-value of 0.05 corresponds to 0.17 (0.05/3).

*mMRC* modified Medical Research Council, *BAE* bronchial artery embolization, *FACED* Forced expiratory volume in 1 s (F), age (A), chronic colonization by *Pseudomonas aeruginosa* (C), extension fo the disease by radiological assessment (E), dyspnea (D), *BSI* Bronchiectasis Severity Index.

^a^There was a significant difference between normal lung function and the obstructive pattern.

^b^There was a significant difference between normal lung function and the restrictive pattern.

^c^There was a significant difference between the obstructive and restrictive patterns.

### Correlation between the modified Reiff score and lung function

**Figure 1:**
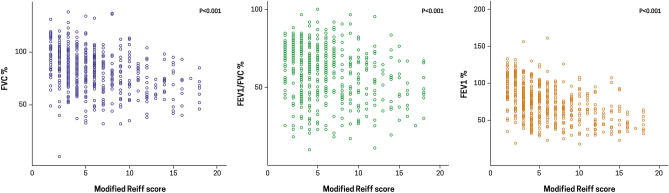
Correlation between the modified Reiff score and lung function. (A) Relationship between the modified Reiff score and FVC %, predicted (B) Relationship between the modified Reiff score and FEV1/FVC % (C) Relationship between the modified Reiff score and FEV1 %, predicted Abbreviations: FVC, forced vital capacity; FEV1, forced expiratory volume in 1 second.

As shown in Fig. 1, the FVC %, FEV_1_/FVC %, and FEV_1_% declined significantly with the elevation in the modified Reiff score (*P* < 0.001 for all). The FEV_1_ declined significantly with the increase in the BSI score (r = − 0.442, respectively; *P* < 0.001) in the obstructive pulmonary disorder group. The FVC decreased significantly with the increase in the BSI score (r = − 0.363, *P* < 0.001) in the restrictive pulmonary disorder group (Supplementary Fig. 1).

## Discussion

To the best of our knowledge, this is the first study to evaluate the clinical characteristics, radiological features, and disease severity according to the spirometric pattern in patients with bronchiectasis. Approximately 52% and 18% patients with bronchiectasis had obstructive and restrictive ventilatory disorders, respectively. The frequency of dyspnea, higher disease severity (including high BSI and FACED scores), higher sputum culture positivity for *Pseudomonas aeruginosa*, and greater radiological severity (i.e., high modified Reiff scores) were predominant in bronchiectasis accompanied by obstructive ventilatory disorder. On the other hand, bronchiectasis with restrictive ventilatory disorder was associated with a high prevalence of NTM disease. There was no significant correlation between the spirometric patterns and the type of bronchiectasis; however, lung function decreased with the increase in the modified Reiff score.

Patients with bronchiectasis frequently experience structural and functional lung damage that vary in severity^2,8,9^. Our notable finding was that more than half of patients (52%) with bronchiectasis showed obstructive ventilatory disorder. In previous studies, approximately up to 35% of bronchiectasis were known to experience obstructive ventilatory disorders^5,12–15^. Bronchiectasis is known to manifest as an obstructive ventilatory disorder due to continuous airway destruction and distortion caused by chronic inflammation and abnormal mucociliary clearance^2^. In our study, the bilateral lung involvement was high at 70% in patients with bronchiectasis. Bilateral lung involvement resulted in widespread inflammation of the bronchial tree and damage to the lung parenchyma, which is believed to have resulted in pulmonary impairment of obstructive ventilatory disorder. Additionally, patients with bronchiectasis may also experience restrictive ventilatory disorder. Bronchiectasis accompanied by atelectasis and pleural disease, parenchymal scarring, and peribronchial fibrosis is thought to be associated with restrictive ventilatory disorder^11^. In contrast with obstructive ventilatory disorder, only a few studies have described restrictive ventilatory disorder and the presumable prevalence was reported to be 8–15% among patients with bronchiectasis^12,13,15^. Our study also reported a similar result, i.e., 18% of patients with bronchiectasis had restrictive ventilatory disorder; however, verification through a large-scale study is necessary since it accounted for a small proportion of the study population.

Our notable finding was that bronchiectasis with obstructive ventilatory disorder was characterized by greater symptoms of dyspnea, disease severity and radiological severity. We measured the degree of dyspnea using the mMRC score, which was significantly higher in bronchiectasis with obstructive ventilatory disorder. mMRC is a subjective measure of dyspnea in COPD, which is known to be associated with the decrease in FEV_1_^16^. Similarly, in our study, the degree of dyspnea as measured by the mMRC score was higher in the obstructive ventilatory disorder group with low FEV_1_. Moreover, we evaluated disease severity using the BSI and FACED scores. The FACED score [FEV_1_% predicted (F), age (A), chronic colonization by *Pseudomonas aeruginosa* (C), extension of the disease by radiological assessment (E) and dyspnea (D)]^9^ is a five-point instrument that predicts the probability of all-cause mortality after 5 years of follow-up, whereas the BSI^8^ is a seven-point scale that identifies patients with bronchiectasis at risk for future mortality, hospitalization, exacerbations, and deterioration in the quality of life. Our study revealed high disease severity and poor prognosis, as measured using the BSI and FACED, in bronchiectasis with obstructive ventilatory disorder. These results may be related to low BMI, low FEV_1_, high mMRC score, and high Pseudomonas aeruginosa infection, which are the clinical features of bronchiectasis with obstructive ventilatory disorder ascertained by our study. Although most studies have reported that the FEV_1_ decreases with the increase in CT severity^11,17,18^, no study has analyzed the relationship between CT severity and lung function in bronchiectasis with obstructive ventilatory disorder. Our study is the first to demonstrate the impairment in lung function, including FVC and FEV_1_, and CT severity using the modified Reiff score in bronchiectasis with obstructive ventilatory disorder. Therefore, these findings emphasize the importance of the management of lung function in bronchiectasis with obstructive ventilatory disorder.

Another interesting finding of our study was that there was no significant correlation between the spirometric pattern and radiological type of bronchiectasis. However, the increase in the radiological severity, which was measured by the modified Reiff score, was accompanied by the increase in the severity of impairment of lung function. Bronchiectasis is known to manifest as a combination of three morphologies, viz., cylindrical, varicose, and cystic^19^. Few studies have investigated the relationship between the type of bronchiectasis and the spirometric pattern. In contrast with our study, other studies have suggested that cylindrical bronchiectasis is associated with obstructive ventilatory disorder^15,20^. However, it is difficult to generalize the results of studies that incorporated a small patient population. Thus, large-scale research on the association between the radiological features of bronchiectasis and lung function is warranted. Our study also showed that the severity of lung function impairment increased with the radiological extent of bronchiectasis. Inflammation and destruction of the lung parenchyma and airways become more severe with the expansion in the extent of bronchiectasis, which may lead to a decrease in lung function. Other studies have also reported similar results^11,17^. Thus, we can infer that the extent of bronchiectasis, rather than the type of bronchiectasis, affects lung function.

This study had several limitations. First, it was conducted at a single center in South Korea. Second, its retrospective and observational design could have introduced potential biases. For example, the proportion of patients with obstructive ventilatory disorder was high in this bronchiectasis population, possibly because this study was conducted in patients with bronchiectasis who underwent pulmonary function testing. Third, the BSI and FACED scores were high in the bronchiectasis with obstructive ventilatory disorder group. FEV_1_ is included in the BSI and FACED evaluation criteria, which may be a confounding factor. However, our study showed that low BMI, high mMRC score, and high prevalence of Pseudomonas aeruginosa infection were clinical features associated with the severity of bronchiectasis with obstructive ventilatory disorder, even after excluding these confounding factors.

In conclusion, among patients with bronchiectasis, 52% had obstructive ventilatory disorder and 18% had restrictive ventilatory disorder. Bronchiectasis with obstructive ventilatory disorder had more dyspnea, and greater disease and radiological severity. The extent of bronchiectasis rather than radiologic type of bronchiectasis affects lung function impairment, however more research is needed.

## Methods

### Study setting and patients population

**Figure 2:**
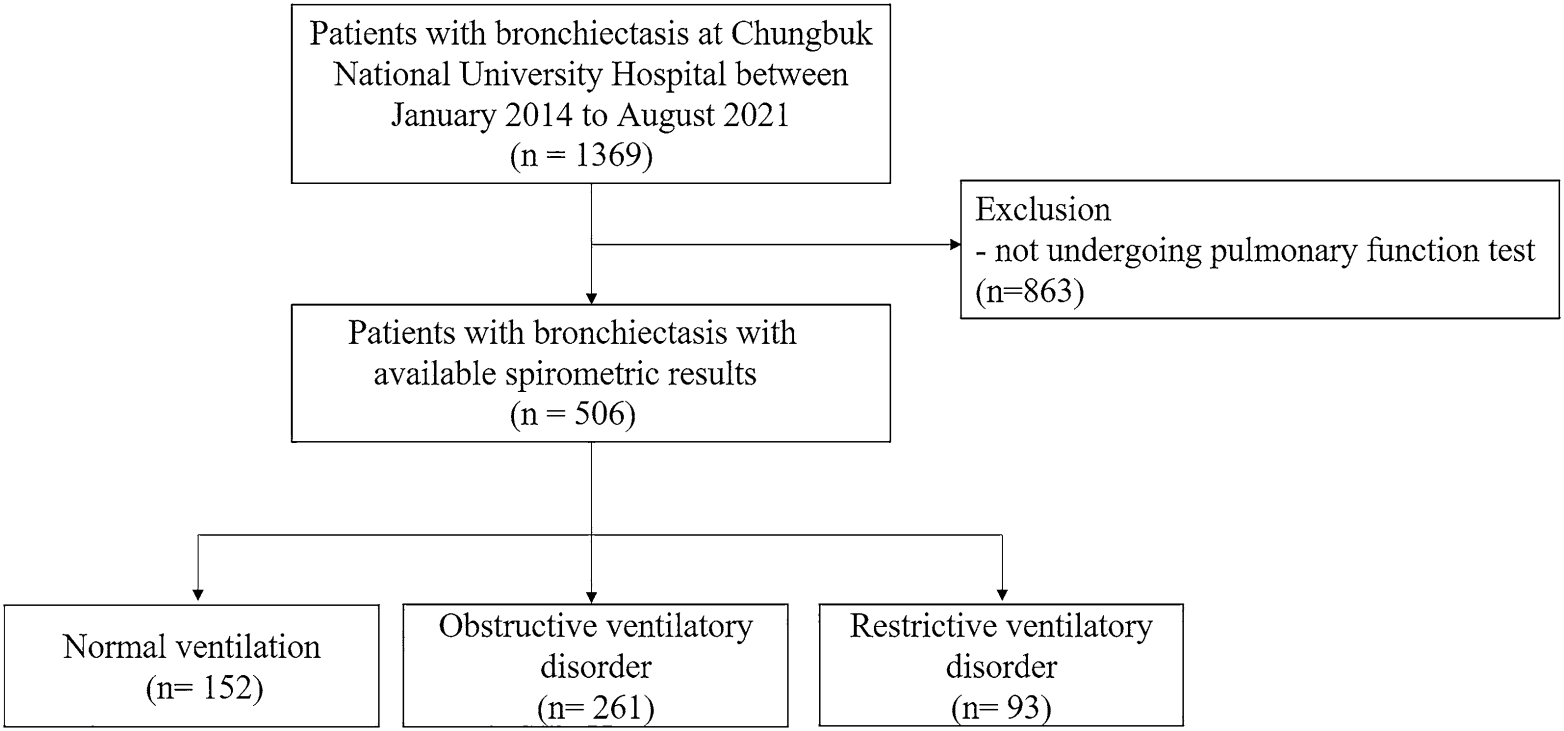
Flow chart of study population.

We retrospectively reviewed the medical records of 1369 patients at Chungbuk National University Hospital (a 793-bed referral hospital) in Cheongju, Republic of Korea, in whom bronchiectasis was diagnosed between January 2014 to August 2021. Of those patients, those not undergoing pulmonary function test (n = 863) were excluded, a total of 506 patients were included in the study. We classified patients with bronchiectasis into three groups according to spirometric patterns (Fig. 2).

### Ethics declarations

The study protocol was approved by the Institutional Review Board of Chungbuk National University Hospital (IRB No. 2021-09-023) and was conducted in accordance with the amended Declaration of Helsinki (as revised in 2013). Patient information was anonymized and de-identified prior to analysis. Therefore, the need for informed consent was waived owing to the retrospective nature of the study.

### Pulmonary function tests

Pre-bronchodilator and post-bronchodilator spirometry was performed according to American Thoracic Society/European criteria^21^. The absolute values of FEV_1_ and forced vital capacity (FVC) were recorded, and the percentage of predicted values for FEV_1_ and FVC were calculated using an automatic calculator with a reference equation obtained from a representative Korean sample^22^. Normal ventilation was defined as post-bronchodilator FEV_1_/FVC ≥ 0.70 and FVC ≥ 80% predicted. Obstructive ventilatory disorder was defined as post-bronchodilator FEV_1_/FVC < 0.70. Restrictive ventilatory disorder was defined as FEV_1_/FVC ≥ 0.7 and FVC < 80% predicted^23^.

### Bronchiectasis severity: radiological and disease severity

The radiological severity of bronchiectasis was measured using the modified Reiff score^24^. Chest computed tomography (CT) was used to evaluate the radiological extent of bronchiectasis in all participants. The number of lobes involved (the lingula was considered to be a separate lobe) and degree of dilatation (tubular: 1, varicose: 2, and cystic: 3) were determined by three researchers, including two pulmonologists (SHK and BY) and one radiologist (JYY), based on the consensual interpretation of the chest CT image. The FACED and BSI scores were calculated to assess the clinical status and severity of the bronchiectasis, based on previous studies^8,9^.

### Microbiology

Spontaneous sputum or lower tract specimens (bronchoalveolar lavage) were obtained from all patients. The specimens were subjected to microbiological analyses according to standard methods^25^. Conventional semi-qualitative bacterial and fungal cultures were performed. All samples underwent initial Gram staining prior to sputum culture, if the Murray and Washington criteria were met^26^. Nontuberculous mycobacteria (NTM) lung disease was diagnosed using the microbiological criteria provided by the American Thoracic Society and Infectious Disease Society of America; (1) two positive sputum cultures, (2) one positive bronchial wash or lavage, (3) compatible mycobacterial histological features such as granulomatous inflammation, and positive results on acid-fast bacilli lung biopsy and/or lung biopsy culture, and (4) more than one positive sputum culture or bronchial wash^27^. Chronic cavitary pulmonary aspergillosis is one or more pulmonary cavities possibly containing one or more aspergillomas with serological or microbiological evidence implicating *Aspergillus* spp. with significant pulmonary symptoms and overt radiological progression over at least 3 months of observation^28^.

### Statistical analysis

Data were presented as the median and interquartile range (IQR) for continuous variables and frequency (percentage) for categorical variables. Continuous variables were compared using the Kruskal–Wallis test and the Pearson chi-squared test or Fisher exact test was used for categorical variables. Moreover, P-values for comparing the three ventilatory disorder groups were adjusted using Bonferroni correction. Spearman's rho correlation method was used to confirm the existence of a correlation between the BSI score and lung function tests. All tests were two-sided, and P-values < 0.05 were considered statistically significant. All statistical analyses were conducted using IBM SPSS Statistics for Windows (version 21.0; IBM Corp., Armonk, NY, USA) and STATA (version 15; Stata Corp., College Station, TX, USA).

## Supplementary Information

Supplementary Information 1. (A) Obstructive ventilatory disorder (B) Restrictive ventilatory disorder

## Data Availability

The data that support the findings of this study are available from the corresponding author upon reasonable request.
