# Supplementary figures and images for: Clinical characteristics, radiological features, and disease severity of bronchiectasis according to the spirometric pattern
Source: Sci Rep. 2022 Aug 1;12:13167. doi: 10.1038/s41598-022-17085-3 (PMC9343368; doi:10.1038/s41598-022-17085-3)

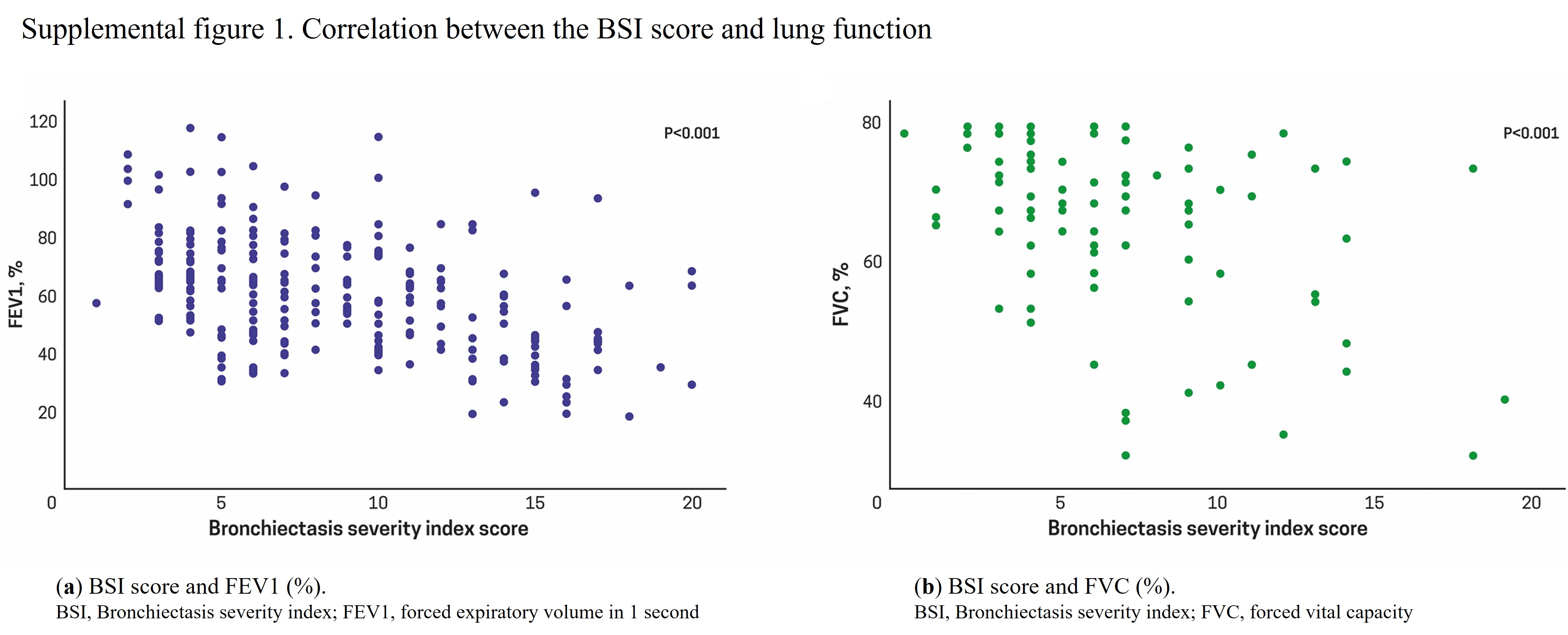

Supplement: Supplementary file 1 — Supplementary Information 1. (A) Obstructive ventilatory disorder (B) Restrictive ventilatory disorder [file 41598_2022_17085_MOESM1_ESM.jpg]
